# Associations between the number of children, age at childbirths and prevalence of chronic low back pain: the Nord-Trøndelag Health Study

**DOI:** 10.1186/s12889-020-09480-0

**Published:** 2020-10-15

**Authors:** Ingrid Heuch, Ivar Heuch, Knut Hagen, Kjersti Storheim, John-Anker Zwart

**Affiliations:** 1 Department of Research, Innovation and Education, Division of Clinical Neuroscience, Oslo University Hospital, P.O. Box 4956, Nydalen, N-0424 Oslo, Norway; 2 Department of Mathematics, University of Bergen, Bergen, Norway; 3 Department of Neuromedicine and Movement Science, NTNU, Norwegian University of Science and Technology, Trondheim, Norway; 4 Clinical Research Unit Central Norway, St. Olavs Hospital, Trondheim, Norway; 5 Research and Communication Unit for Musculoskeletal Health (FORMI), Oslo University Hospital, Ullevål, Oslo, Norway; 6 Faculty of Health Sciences, Oslo Metropolitan University, Oslo, Norway; 7 Faculty of Medicine, University of Oslo, Oslo, Norway

**Keywords:** Low back pain, Childbirth, Delivery, Parity, Musculoskeletal disorder, Epidemiology, HUNT

## Abstract

**Background:**

Associations between childbirths and subsequent risk of low back pain (LBP) have not been clarified. Changes in sex hormone levels or lumbar posture during pregnancy may have an impact on LBP later in life. The purpose of this study was to explore associations between the number of childbirths, age at childbirths and prevalence of chronic LBP in a general population of women.

**Methods:**

Data were obtained from the Norwegian community-based Nord-Trøndelag Health Study, HUNT2 (1995–1997). Women aged 20–69 years indicated whether they suffered from chronic LBP, defined as LBP persisting at least 3 months continuously during last year. Information about LBP was collected from 3936 women who had experienced no childbirths, 3143 women who had delivered one child only and 20,584 women who had delivered 2 or more children. Of these, 7339 women reported chronic LBP. The 595 women who were pregnant when information was collected were considered separately, regardless of previous births, with 80 women reporting chronic LBP. Associations with prevalence of chronic LBP were examined by generalised linear modelling with adjustment for potential confounders in a cross-sectional design.

**Results:**

Women who had delivered one child only showed a higher prevalence of chronic LBP than women with no childbirths (prevalence ratio (PR) 1.11; 95% CI: 1.01–1.22). Among women with one or more childbirths, no overall change in prevalence could be demonstrated with an increasing number of children in analyses adjusted for age at first delivery. In women with at least two childbirths, an age less than 20 years at first childbirth was associated with an increased prevalence of chronic LBP (PR 1.36; 95% CI: 1.25–1.49; compared with age 25–29 years). No association was observed between age at last delivery and chronic LBP. The lowest prevalence of chronic LBP was found among women who were currently pregnant (PR 0.80; 95% CI: 0.63–1.00; compared with women with no childbirths).

**Conclusions:**

Having experienced at least one childbirth seems to be associated with a higher prevalence of chronic LBP later in life. A young age at first childbirth is also associated with a long-lasting increased prevalence.

## Background

Low back pain (LBP) often occurs in women during pregnancy [1, 2]. It has been hypothesised that such pregnancy-related LBP may be related to changes in lumbar posture, perhaps combined with stretching of abdominal muscles or particular hormonal influences in pregnancy [3]. It is not clear, however, whether going through a pregnancy increases a woman’s long-term risk of LBP. Such long-lasting effects may be related to more general hormonal or mechanical changes associated with pregnancies [4]. In most populations, women appear to have a higher prevalence of LBP than men [5], and this difference can possibly be ascribed to reproductive factors.

Some studies have shown a positive relationship between the number of full-term pregnancies a woman has experienced or the total number of children and subsequent prevalence of LBP [4, 6–8]. However, a lower prevalence among women with many children has also been observed [9]. In contrast, a large population-based cross-sectional study [10] found an increased prevalence of LBP among women who had ever been pregnant, although no trend was seen according to the number of pregnancies.

Relationships between maternal age at pregnancies and subsequent risk of LBP have hardly been considered, although a young age at first childbirth was associated with a higher prevalence of LBP in two studies [4, 10]. One study [11] found a difference according to current age, with more frequent back pain in young women with at least one child, but with no similar contrast among middle-aged women. Other studies have shown differences in occurrence of LBP during various stages of pregnancy according to the number and characteristics of previous pregnancies [12], but the mechanisms responsible for this kind of pain may differ from those causing LBP in nonpregnant women.

The purpose of the present study was to assess associations between childbirths and chronic LBP in a community-based Norwegian study of women. We addressed the following specific questions. Does the prevalence of chronic LBP depend on the number of childbirths a woman has experienced? Does a woman’s age at first or last (most recent) childbirth affect the prevalence of chronic LBP? Do any potential relationships of this kind depend on the current age of the woman?

Data derived from the same survey have previously been used to study body mass index (BMI) [13] and lipid levels [14] in association with prevalence of chronic LBP, applying a cross-sectional design. Associations have also been studied between physical activity in leisure time [15], body height [16], measures of body size [17] and risk of chronic LBP using a prospective design.

## Methods

### Study design

In the period 1995 to 1997 the whole population of Nord-Trøndelag county in Norway aged at least 20 years received an invitation to participate in the second Nord-Trøndelag Health Study, HUNT2 [18], and were asked to fill in a questionnaire. One question was expressed in this way: “During the last year, have you suffered from pain and/or stiffness in your muscles and joints that has lasted for at least 3 consecutive months?ˮ Each participant answering yes was given the following question: “Where did you have these complaints?” Several body regions were listed. Individuals answering yes to the first question and including the lower back as a relevant region were regarded as having chronic LBP [19].

The participants were asked to give information on duration of education, physical activity in leisure time and smoking, and information needed for determining Hospital Anxiety and Depression Scale (HADS) scores [20]. All women were asked to indicate how many children they had given birth to, and how old they were when they experienced their first and last (most recent) childbirth. The women also indicated whether they were pregnant at the time when the questionnaire was filled in. A clinical examination with measurements of body weight and height was performed.

The target population of the HUNT2 study comprised 37,503 women in the age range 20–69 years. Of these, a total of 28,520 women participated in HUNT2 [18]. Information about presence or absence of chronic LBP was collected from 3936 women without any childbirths, 3143 women with one childbirth only and 20,584 women with at least 2 childbirths. In our present study, the additional 595 women with information on LBP who were pregnant at the time of HUNT2 were considered as a separate category, regardless of previous childbirths. In total 28,258 women were included in the study, corresponding to a participation rate of 75.3%.

### Categorisation of variables

Current age in HUNT2 was categorised into five 10-year intervals in the statistical analyses. BMI, defined as weight/height^2^ and computed in kg/m^2^, was subdivided into three groups: < 25, 25–29.9, ≥30. Categories of education were defined according to duration, ≤9, 10–12, and ≥ 13 years. Cigarette smoking was described using the categories current daily smoking, previous daily smoking and never daily smoking. For physical activity in leisure time, including going to work, one category comprised those engaged in light activity only or hard physical activity (leading to sweating or being out of breath) < 1 h per week. Other categories represented hard physical activity 1–2 and ≥ 3 h per week. The information about physical activity collected in HUNT2 was verified by a reliability and validity study of a subsample [21]. Anxiety and depression were measured by total Hospital Anxiety and Depression Scale (HADS) scores [20], categorised into 5 intervals: 0–4, 5–9, 10–14, 15–19 and ≥ 20.

To maintain confidentiality, women with 9 or more children were combined with those reporting 8 children in the data file considered in the present study. This did not affect the grouping used in the main analysis, corresponding to 0, 1, 2, 3, 4, 5 and ≥ 6 children. Age at a woman’s first childbirth was divided into five age groups computed in years: ≤19, 20–24, 25–29, 30–34, ≥35, and similarly for the age groups at last birth: ≤24, 25–29, 30–34, 35–39, ≥40.

### Data analysis

Associations between the number of children or maternal age at first or last childbirth and relevant covariates were explored by computing the mean number of children or mean age at childbirth, overall and within broad categories of each covariate considered. A more detailed description of the associations was made by tabulating percentages of covariate categories within categories for the number of children and for age at first childbirth among women with 2 or more children.

Associations with prevalence of chronic LBP were evaluated by generalised linear modelling for binomially distributed data with a log-link, with the number of children or maternal age at first or last childbirth as predictor variables, including adjustment for potential confounders. These analyses produced estimates of the prevalence ratio (PR) of LBP relative to a particular reference category. This parameter represents the probability of experiencing LBP in a certain category of the predictor variable, expressed relative to the probability in the reference category, and thus corresponds to relative risk in a prospective study. Initial analyses incorporated adjustment only for current age at the time when information was collected in HUNT2. Additional adjustment was then introduced for other potential risk factors as BMI, duration of education, physical activity in leisure time and smoking, and, where appropriate, other factors related to the childbirths. As adjustment for age at last childbirth could only be carried out among women with 2 or more children, separate analyses were performed among women with a single childbirth only.

In the main analyses, variables adjusted for were regarded as categorical. Separate *P*-values were computed for linear trends in the associations, considering the number of children or age at childbirths as continuous predictor variables. These analyses were based on the original one-year values of age at childbirths. Linearity was checked by computing *P*-values for additional quadratic terms. Particular tests were performed for interaction between the number of childbirths or age at childbirths and each factor adjusted for. Because of the special interest in a potential interaction with current age, separate analyses of associations with the number of childbirths and age at first childbirth were also carried out within 10-year categories of current age.

A minor part of the data set had missing covariate values, and each adjusted analysis was carried out including women with complete information only. The number of women considered in each case is shown in the tables. Overall HADS scores were available only for 78.3% of the women included. To avoid losing a relatively large part of the data, additional adjustment including HADS score was only performed in separate sensitivity analyses.

All statistical analyses were carried out using IBM SPSS Statistics version 25 (IBM).

## Results

Women with an education of short duration tended to have more children than other groups (Table 1). Similar but weaker increases in the number of children were observed among women with BMI ≥ 25, among those with a large HADS score, women reporting little physical activity and among former smokers. Mean age at childbirths showed only relatively minor differences over categories of covariates, except for current smokers with rather low mean values and women with an education of long duration with large values (Table 1). Consistent associations were found considering percentages of covariate values within categories of number of children or age at first childbirth (Additional file 1: Supplementary Tables 1 and 2).

**Table 1:** Mean number of childbirths and mean age at childbirths, in broad subgroups defined by covariates

	Mean number of childbirths	Mean age at childbirth among women with 1 child only	Mean age at first childbirth among women with ≥2 children	Mean age at last childbirth among women with ≥2 children
Total data set^a^	2.3	24.9	22.6	29.6
Age when information was collected (years)				
20–29	0.8	22.7	21.0	24.8
30–39	2.2	25.9	22.8	28.9
40–49	2.4	25.6	22.4	29.1
50–59	2.7	26.1	22.5	29.5
60–69	3.0	26.4	23.3	32.5
BMI (kg/m^2^)				
< 25	2.0	24.6	22.7	29.1
25–29.9	2.4	25.3	22.6	29.7
≥ 30	2.6	25.4	22.5	30.2
Physical activity per week				
< 1 h hard	2.3	25.1	22.6	29.5
1–2 h hard	1.9	24.5	22.9	29.0
≥ 3 h hard	1.6	24.4	22.6	28.7
Cigarette smoking				
never	2.1	25.8	23.3	30.2
daily former	2.4	25.5	22.8	29.8
daily current	2.2	23.7	21.6	28.5
Education (years)				
≤ 9	2.8	24.8	21.8	29.9
10–12	2.0	24.2	22.3	28.6
≥ 13	1.8	26.6	24.8	30.6
HADS score				
0–4	2.1	25.0	22.8	29.3
5–9	2.2	24.8	22.7	29.4
10–14	2.3	25.0	22.5	29.5
15–19	2.4	24.3	22.2	29.3
20–39	2.5	24.2	21.9	29.5

^a^Among women who were not pregnant when information was collected

Women who had delivered one child only showed an 11% higher estimated prevalence of chronic LBP than women with no childbirths, after adjustment for BMI, physical activity, education and smoking (Table 2). A tendency to an increasing prevalence with more childbirths after the first was seen in initial analyses, at least among women with 1–4 births, but mainly disappeared after adjustment for age at first childbirth (Table 2). The lowest prevalence of chronic LBP was observed among women who were pregnant when information was collected, with a reduction in prevalence after additional adjustment of about 20% compared with non-pregnant women who had not experienced any childbirths (Table 2).

**Table 2:** Prevalence of chronic LBP by number of childbirths and pregnancy status

	Total number of women in category	Number of women with chronic LBP (%)	PR (95% CI) with adjustment for age only	PR (95% CI) with additional adjustment^a^	PR (95% CI) with additional adjustment^a^ including age at first childbirth^b^
Number of women included in the analysis			28,258	25,444	21,277
Number of childbirths					
0	3936	654 (16.6)	1.00 (reference)	1.00 (reference)	
1	3143	733 (23.3)	1.17 (1.06–1.29)	1.11 (1.01–1.22)	1.00 (reference)
2	9210	2460 (26.7)	1.18 (1.09–1.29)	1.12 (1.03–1.22)	1.02 (0.94–1.10)
3	7147	2096 (29.3)	1.23 (1.13–1.33)	1.17 (1.07–1.27)	1.03 (0.95–1.12)
4	2730	912 (33.4)	1.32 (1.20–1.45)	1.21 (1.10–1.34)	1.06 (0.96–1.16)
5	958	312 (32.6)	1.25 (1.11–1.41)	1.11 (0.97–1.27)	0.96 (0.85–1.09)
≥ 6	539	172 (31.9)	1.21 (1.05–1.40)	1.07 (0.91–1.27)	0.91 (0.77–1.07)
Currently pregnant^c^	595	80 (13.4)	0.86 (0.69–1.06)	0.80 (0.63–1.00)	
P for categorical effect			< 0.001^d^	0.008^d^	0.29
P for linear trend			0.001^b^	0.08^b^	0.70

^a^Adjustment for age, BMI, physical activity, education, smoking

^b^Among women with at least one child

^c^Regardless of the number of previous children

^d^Among all women who are not currently pregnant

Among women with a single child, the age at childbirth showed a non-linear relationship with the prevalence of chronic LBP (Table 3), possibly with an approximate U-shape. This relationship was maintained after adjustment for potential confounders. Compared with women experiencing childbirth in the age interval 25–29 years, those with a childbirth before age 20 years had an estimated increase in prevalence of 42% (Table 3). The estimates also suggested that women with a single childbirth at age ≥ 30 years might have a higher prevalence than those who experienced the childbirth at age 25–29 years.

**Table 3:** Prevalence of chronic LBP by age at childbirth, in women with one childbirth only

	Total number of women in category	Number of women with chronic LBP (%)	PR (95% CI) with adjustment for age only	PR (95% CI) with additional adjustment^a^
Total number of women in the analysis			3143	2893
Age at childbirth (years)				
≤ 19	420	119 (28.3)	1.50 (1.23–1.83)	1.42 (1.16–1.74)
20–24	1257	288 (22.9)	1.22 (1.03–1.43)	1.14 (0.96–1.35)
25–29	927	182 (19.6)	1.00 (reference)	1.00 (reference)
30–34	361	98 (27.1)	1.18 (0.96–1.47)	1.17 (0.94–1.45)
≥ 35	178	46 (25.8)	1.07 (0.80–1.42)	1.14 (0.86–1.52)
P for categorical effect			0.002	0.027
P for linear trend			0.005	0.12
P for quadratic effect			0.028	0.040

^a^Adjustment for age, BMI, physical activity, education, smoking

Women with at least two childbirths showed a clear non-linear U-shaped relationship between age at first childbirth and prevalence of chronic LBP (Table 4). This relationship persisted after adjustment for potential confounders, including age at last childbirth. In comparison to women aged 25–29 years at first childbirth, women with a first childbirth at age ≤ 19 years had a 36% increase in estimated prevalence of LBP, and those with a first childbirth at age ≥ 35 had a 23% increase (Table 4). In contrast, age at last childbirth showed no definite association with prevalence of chronic LBP (Table 4).

**Table 4:** Prevalence of chronic LBP by age at first and last childbirths, in women with at least two childbirths

	Total number of women in category	Number of women with chronic LBP (%)	PR (95% CI) with adjustment for age, number of children, and age at first or last childbirth	PR (95% CI) with additional adjustment^a^
Number of women included in the analysis			20,584	18,384
Age at first childbirth (years)				
≤ 19	4020	1397 (34.8)	1.52 (1.41–1.65)	1.36 (1.25–1.49)
20–24	11,099	3278 (29.5)	1.27 (1.19–1.35)	1.18 (1.10–1.27)
25–29	4560	1057 (23.2)	1.00 (reference)	1.00 (reference)
30–34	772	179 (23.2)	0.98 (0.85–1.12)	1.04 (0.89–1.20)
≥ 35	133	41 (30.8)	1.25 (0.95–1.63)	1.23 (0.91–1.66)
P for categorical effect			< 0.001	< 0.001
P for linear trend			< 0.001	< 0.001
P for quadratic effect			< 0.001	< 0.001
Age at last childbirth (years)				
≤ 24	3082	956 (31.0)	1.04 (0.96–1.12)	1.03 (0.94–1.11)
25–29	7736	2215 (28.6)	1.00 (0.95–1.06)	1.01 (0.95–1.07)
30–34	6445	1798 (27.9)	1.00 (reference)	1.00 (reference)
35–39	2843	847 (29.8)	1.03 (0.96–1.11)	1.05 (0.97–1.14)
≥ 40	478	136 (28.5)	0.96 (0.82–1.12)	0.98 (0.82–1.17)
P for categorical effect			0.66	0.73
P for linear trend			0.46	0.74
P for quadratic effect			0.96	0.82

^a^Adjustment for age, number of children, age at first or last childbirth, BMI, physical activity, education, smoking

The relative prevalence of LBP among women with a single child, compared with those with no children, depended on the current age at the time when information was collected (Table 5). From an age of about 40 years the increased prevalence among women with one child disappeared. Among women with current age 20–29 years, each additional childbirth after the first also seemed to be associated with an increased prevalence (Table 5), but no corresponding trend was seen in other intervals of current age. The associations with age at first childbirth among women with ≥2 children were quite similar in all intervals of current age, at least until an age of about 60 years (Table 5).

**Table 5:** Prevalence of chronic LBP by reproductive factors in different strata of age

	PR (95% CI) for 1 childbirth vs. no childbirth^a^	PR (95% CI) per child, among women with ≥1 childbirth^b^	PR (95% CI) for age at first childbirth ≤19 vs. 25–29, among women with ≥2 childbirths^c^	PR (95% CI) for age at first childbirth ≥35 vs. 25–29, among women with ≥2 childbirths^c^
Age when information was collected (years)				
20–29	1.32 (1.07–1.62)	1.19 (1.06–1.33)	1.49 (0.77–2.90)	
30–39	1.15 (0.91–1.44)	0.99 (0.93–1.05)	1.27 (1.05–1.53)	1.12 (0.33–3.83)
40–49	0.99 (0.81–1.20)	1.04 (1.00–1.09)	1.46 (1.24–1.73)	1.42 (0.89–2.27)
50–59	0.99 (0.79–1.25)	1.00 (0.97–1.04)	1.46 (1.24–1.72)	1.47 (0.89–2.43)
60–69	0.81 (0.62–1.05)	0.95 (0.91–0.98)	1.21 (0.99–1.48)	0.82 (0.42–1.62)
P for interaction with categorical age	0.020	0.002	0.10^d^	
P for interaction with linear age	0.001	0.002	0.75^d^	

^a^Adjustment for BMI, physical activity, education, smoking

^b^Adjustment for BMI, physical activity, education, smoking, age at first childbirth

^c^Adjustment for BMI, physical activity, education, smoking, number of childbirths, age at last childbirth

^d^Based on likelihood ratio test for interaction with complete quadratic relationship with age at first childbirth

In tests for interaction with other covariates, a significant result was only obtained for duration of education and the difference in prevalence of LBP between women with one child and no children (*P* = 0.014). In this situation, the PR associated with having a single child compared to no children was 0.88 (95% CI: 0.74–1.05) among women with a duration of education ≤9 years, 1.08 (95% CI: 0.93–1.25) with a duration of 10–12 years, and 1.29 (95% CI: 1.04–1.60) with a duration ≥13 years.

In separate sensitivity analyses with additional adjustment for HADS, associations with the number of children (Additional file 1: Supplementary Table 3) or the age at first childbirth among women with ≥2 children (Additional file 1: Supplementary Table 4) deviated to some degree from the main results. In particular, it was no longer evident that women with a first childbirth at age ≥ 35 years had a higher prevalence than those aged 26–30 years at first childbirth. Because of missing values for HADS, the number of women included was considerably lower in these analyses. To assess the actual effect of adjustment for HADS, an analysis was also performed without adjustment for HADS including exactly the same set of women. The adjustment led only to minor changes in risk estimates (Additional file 1: Supplementary Tables 3, 4), both for the number of children and age at first childbirth.

## Discussion

In this study, the most striking result was an increased prevalence of chronic LBP in women with a young age at first childbirth. This association seemed to last for a period of at least 40 years after the relevant childbirth. On the other hand, age at last birth was not related to the prevalence of LBP. Women who had experienced at least one childbirth showed a moderate increase in prevalence of chronic LBP compared with those without children. This difference lasted until an age of about 40 years. Additional childbirths after the first did not appear to affect the prevalence, except perhaps among women aged 20–29 years. The prevalence estimate among women who were pregnant when information was collected was lower than estimates among non-pregnant women.

### Strengths and limitations

The basic design of this study has several strengths. The target population represented all female residents of a Norwegian county within the relevant age interval, and the participation rate was relatively high. The ethnic background of the population was nearly uniform and overall socioeconomic differences were small [18]. The composition of the population was in many ways similar to that of the overall Norwegian population but did not include any large towns. Although a cross-sectional study design was used, information concerning pregnancies mostly related to events several years before the LBP occurred. In this sense the study design was more similar to a longitudinal one.

The information collected on potential risk factors, mainly based on self-reports, should be quite reliable, in particular for the number of children and age at childbirths. Although the number of children considered was truncated above at the value 8, this did not influence the categorical variable included in the main statistical analyses. However, the mean number of children as shown in Table 1 is not completely accurate, but since only 0.2% of the women indicated that they had 8 or more children, the discrepancy must be minor. The information used in this study did not specify whether a woman had experienced multiple births, and in such cases the number of children does not represent the number of pregnancies. Thus we are not able to study associations with parity in the ordinary sense, but this difference is probably of minor importance when results are compared across studies. Moreover, for women with 3 or more children, associations with the age at childbirths between the first and last ones could not be considered.

The information about chronic LBP was also based on self-reports and only referred to the last year before the questionnaire was returned. Specific information was not available on LBP occurring during pregnancies or shortly afterwards, and there was no information on pain intensity. The definition of chronic LBP applied may in particular be problematic for women who were pregnant when information was collected, as it was not known how far each woman had progressed in the pregnancy. Thus the LBP reported may partly reflect the situation before the pregnancy.

### Previous studies

The most comprehensive study of associations between pregnancies and subsequent occurrence of LBP is the Dutch population-based cross-sectional study of Wijnhoven et al. [10], including 11,428 women aged 20–59 years. Analyses were carried out separately for three mutually exclusive pain categories, combined chronic LPB and chronic upper extremity pain (UEP), chronic LBP only and chronic UEP only. Thus specific results are not directly comparable to our estimates, but considering adjusted associations reported from the first two sets of analysis, the main findings are very similar to those obtained here. Ever-pregnant women had a slightly higher prevalence of LBP than never-pregnant, but no further increase in prevalence was found with a larger number of children. An age at first delivery less than 20 years was associated with a substantially increased prevalence, with a moderate increase also in the 20–25-year interval. The prevalence among women who were pregnant when the study was carried out was rather low.

Other studies of associations between pregnancies and later prevalence of LBP have mostly used considerably broader definitions of the disorder. Thus the population-based study of Silman et al. [4] in the United Kingdom, comprising 2617 women, included any experience of LBP persisting for more than 24 h. In this study, the prevalence of LBP was similar among women with no children and those with one child, but then increased steadily for women with 2, 3 and ≥ 4 children. Surprisingly, a slightly stronger increase in prevalence of LBP with regard to the number of children was observed among men, even after adjustment for multiple confounders [4]. The parallel associations found in males and females were interpreted as an effect of childrearing.

Several other studies, partly based on different designs and smaller data sets, have shown positive associations between the number of children or pregnancies and the general prevalence of LBP in women [6, 7, 22–24]. However, there are also studies indicating no relationship with prevalence of back pain [25] or even an inverse association [9]. Most studies did not take potential confounders into account [6, 23] or included adjustment for age only [7, 24]. The number of children or pregnancies was categorised in various ways, sometimes dichotomising only [9, 22, 23]. Although different definitions were used for back pain, most studies imposed few limitations on the duration of pain. Thus the classification did not correspond to chronic LBP as considered here, and the recorded frequency of back pain was in several cases considerably larger.

One study [8] found a positive association between the number of live births and occurrence of LBP only among women aged 50 years or more. Another study [11] compared women with at least one child to those with no children and found more frequent back pain among parous women aged 18–25 years but not among those aged 45–50 years. Age at first delivery or pregnancy has apparently been considered as a potential risk factor for LBP only in the studies by Silman et al. [4] and Wijnhoven et al. [10], both finding a greater prevalence of LBP among women with a first delivery or pregnancy before age 20 years.

Other kinds of studies have focused on possible associations between the number of previous childbirths or pregnancies and back pain occurring during a particular later pregnancy. Some studies [1, 26] have found a general positive association of this kind, while others [12, 27] simply reported a larger prevalence of back pain among women with at least one previous pregnancy. Still other studies [2, 28] found no such relationship. Several studies found a greater risk of back pain during pregnancy in very young women [2, 27, 29] or more intense pain [2, 26].

### Interpretation

It has been hypothesised that back pain diagnosed during pregnancy largely represents a different medical entity from back pain experienced in other periods of a women’s life [26]. Alternatively, the two kinds of pain may essentially reflect the same underlying disorder [30], although risk factor levels may be different in the two situations. As an additional complication, pelvic girdle pain has been combined with LBP in most epidemiological studies of pain during pregnancy [31]. For these reasons, it may not be appropriate to compare our results to results from studies dealing with LBP in pregnancy or shortly afterwards, although some of the mechanisms responsible for the pain may still be similar. The low prevalence found in our study among women who were pregnant when information was collected is probably related to the definition of chronic LBP, requiring the presence of pain for 3 consecutive months.

Considering other studies of associations with general prevalence of LBP, the most important difference is that no increased prevalence was found by us with an increasing number of children after the first one, except possibly among women aged 20–25 years. To some extent, this difference can probably be explained by the wider definition of LBP used in the majority of other studies. Strictly speaking, only Wijnhoven et al. [10] applied a comparable definition of chronic LBP. In addition, the adjustment carried out for potential confounders was less complete in most other studies. In our data, a positive association with the number of children was initially observed with partial adjustment only, but the association disappeared in the final model. A similar change occurred in the analysis of the data of Wijnhoven et al. [10].

Duration of education was used in our analysis as an indicator of socioeconomic status. More detailed information based on variables such as income was not available. In particular for the association observed with early age at first birth, it is possible that residual confounding by socioeconomic status still plays a certain role, even after the adjustments made. Women with a long duration of education had a lower prevalence of LBP and tended to experience their first childbirth at a late age (Additional file 1: Supplementary Table 2). If this is representative of high socioeconomic levels more generally, more accurate adjustment for socioeconomic status might further reduce the PR estimate for an early age at first birth. Residual confounding may also affect the association indicated comparing women with a single child with those with no children. The significant interaction found with education for this variable may represent a chance finding among the large number of tests carried out for interaction, but could also reflect an underlying true heterogeneity. HADS score as an indicator of mental status was not available for a relatively large part of the data set, and it is not clear whether the individuals with known score represented a random subsample. However, adjustment for HADS among individuals with known values had no major influence on risk estimates.

Similar results were obtained for the association between LBP prevalence and age at first birth considering women with a single child and of those with 2 or more children. The non-linear relationship approached a U-shape, although the estimated increase in prevalence among women with a first delivery at age ≥ 35 years was not of the same magnitude as among those with a first delivery at age ≤ 19 years, and the PR estimate in the top category of age at first delivery dropped after adjustment for HADS score. Wijnhoven et al. [10] also found the lowest LBP prevalence in the interval 25–29 years for age at first delivery, with similar results over categories of current age.

Our detailed statistical analysis included as predictor variables both a woman’s age when information was collected and her age at first childbirth. A standard analysis cannot additionally include time since first childbirth as a predictor because of linear dependence between the three variables [32]. Although this problem may be circumvented introducing further modelling assumptions [32], we have chosen the simpler approach focusing on age and at age at first childbirth only. This is also consistent with the modelling approach of Wijnhoven et al. [10]. A real association with time since childbirth will then be expressed as an interaction between age and the variable representing one childbirth [32]. The results shown in Table 5 for women with a single childbirth compared to those with no children, indicating such an interaction, can thus also be interpreted as an effect of time since first childbirth, with the prevalence of LBP gradually decreasing in the period after the childbirth.

### Mechanisms

Although our study is based on a cross-sectional design, it is still possible to discuss what kind of potential mechanisms underlying LBP may be consistent with our results. Only mechanisms that account for a moderate medium-term harmful effect of going through a first pregnancy are relevant in the present context. Even more important, the detrimental long-term effect of experiencing the first pregnancy at a young age should be accounted for.

Two main types of mechanism have been considered, based on hormonal effects of going through a pregnancy or mechanical effects. It has been hypothesised that a hormonal influence on soft tissues supporting the spine may play an essential role [4], possibly leading to laxity of joints and ligaments [33]. Increased estrogen levels may thus produce a higher risk of LBP during and after pregnancy, perhaps with the hormone relaxin as an intermediate step [33]. This hypothesis is consistent with the higher risk of LBP observed among women using hormone replacement therapy [10] or oral contraceptives [10]. Ligament laxity reaches higher levels in particular in the second and third trimesters of pregnancy [34] and appears to be associated with back pain with onset during pregnancy, lasting for some time afterwards [35]. Essential changes of this kind occurring during the first pregnancy in a woman’s life seem to offer the best explanation of the associations found in the present study, although it is not entirely clear that the effects would be long-lasting. The higher risk of LBP among women with an early first delivery may possibly be ascribed to young women being particularly sensitive to hormonal changes in estrogen and relaxin [27], with a more pronounced collagen laxity [27]. Estrogens may also act as modulators in the processing of pain [36], but such effects of hormonal changes in pregnancy would hardly be long-lasting.

Purely mechanical effects of childbearing on the risk of LBP may reflect the external burden of lifting and carrying young children [4]. However, the effect would not in most cases be expected to last for decades after childbirths, and the strain should increase with the number of children [8]. Otherwise, biomechanical effects of a pregnancy may be expressed as stress on the spine [8] or direct pressure from the enlarging uterus [4]. The effect of a teenage pregnancy on the developing spine [4] might in particular account for a higher risk of LBP in women with an early first delivery. Biomechanical effects may in general be related to changes in posture during various parts of the pregnancy [3]. However, no clear picture has emerged relating such changes to LBP, and most changes are not long-lasting [3]. Moreover, pregnancy-related back pain occurs so early in pregnancy that mechanical factors are less relevant [37], although the implications for long-term LBP are less clear. Overall, mechanical explanations seem less convincing than those based on hormonal effects.

It has also been suggested that use of epidural anesthesia during labour may increase the risk of subsequent back pain [38], although this is not supported by randomised trials [39]. Such effects might be related to postural problems at delivery exacerbated by muscular relaxation [38], but this seems less likely to produce pain lasting several years after delivery. Finally, it is possible that musculoskeletal disease conversely affects reproduction [40], but it is difficult to see how the particular associations observed in the present study could be generated in this way.

More than 40 years ago, Mantle et al. [41] considered back pain that mostly occurred in connection with the first pregnancy in a woman’s life. It was suggested that the pain might be related to some connective tissue, perhaps ligamentous, giving way during the first pregnancy or delivery and remaining with the woman for the rest of her life. Regardless of the triggering mechanism involved, this would be consistent with the results of the present study, especially if young pregnant women were particularly vulnerable.

## Conclusions

Women with at least one childbirth have a higher prevalence of chronic LBP later in life than women without children. Among women with children, experiencing the first childbirth at an age < 25 years is associated with an increased prevalence of chronic LBP.

## Supplementary information

**Additional file 1: Supplementary Table 1.** Percentage of women in each covariate category, by number of childbirths. **Supplementary Table 2.** Percentage of women in each covariate category, by age at first childbirth, among women with at least two childbirths. **Supplementary Table 3.** Prevalence of chronic LBP by number of childbirths and pregnancy status, with and without adjustment for HADS. **Supplementary Table 4.** Prevalence of chronic LBP by age at first childbirth, in women with at least two childbirths, with and without adjustment for HADS.

## Data Availability

The data set analysed belongs to a third party, the HUNT study (the Nord-Trøndelag Health Study). The authors of the current manuscript are not affiliated with the project as such, but have been given permission to analyse the data after obtaining the necessary Norwegian permits. Because of the confidentiality requirements according to Norwegian law, a data set of this kind with information from a complete county at the individual level cannot be made public. However, research groups wishing to analyse data from the HUNT study may apply to the HUNT organisation (http://www.ntnu.edu/hunt) to get access to the data, after having obtained the permits needed according to Norwegian law.

## Abbreviations

BMI: Body mass index
CI: Confidence interval
HADS: Hospital anxiety and depression scale
HUNT: Nord-Trøndelag Health Study
LBP: Low back pain
PR: Prevalence ratio
UEP: Upper extremity pain
