# Supplementary material for: Associations between the number of children, age at childbirths and prevalence of chronic low back pain: the Nord-Trøndelag Health Study
Source: BMC Public Health. 2020 Oct 15;20:1556. doi: 10.1186/s12889-020-09480-0 (PMC7565361; doi:10.1186/s12889-020-09480-0)
Supplement: Supplementary file 1 — Additional file 1: Supplementary Table 1. Percentage of women in each covariate category, by number of childbirths. Supplementary Table 2. Percentage of women in each covariate category, by age at first childbirth, among women with at least two childbirths. Supplementary Table 3. Prevalence of chronic LBP by number of childbirths and pregnancy status, with and without adjustment for HADS. Supplementary Table 4. Prevalence of chronic LBP by age at first childbirth, in women with at least two childbirths, with and without adjustment for HADS. [file 12889_2020_9480_MOESM1_ESM.pdf]

Supplementary Table 1 Percentage of women in each covariate category, by number of childbirths

Number of childbirths	0	1	2	3	4	5	≥6	Currently pregnant
Total number of women	3936	3143	9210	7147	2730	958	539	595
BMI (kg/m^2)								
< 25	58	52	51	44	36	29	25	46
25-29.9	29	33	35	39	42	42	43	40
≥ 30	13	15	14	17	22	29	33	14
Physical activity per week								
< 1hour hard	60	75	76	79	84	88	89	76
1-2 hours hard	25	18	18	16	13	9	6	18
≥ 3hours hard	15	7	6	5	4	3	5	5
Cigarette smoking								
never	58	36	37	40	41	40	43	51
daily former	14	26	28	28	28	29	26	28
daily current	28	38	35	32	31	31	31	21
Education (years)								
≤ 9	15	25	30	38	55	70	77	8
10-12	49	50	48	42	31	23	17	57
≥ 13	36	25	22	21	14	7	6	36
HADS score								
0-4	42	36	35	33	30	33	27	43
5-9	36	34	35	36	36	30	37	39
10-14	15	18	17	19	21	21	20	12
15-19	5	8	8	7	8	11	10	4
20-39	2	4	5	5	6	6	6	2

HADS: Hospital anxiety and depression scale

Supplementary Table 2 Percentage of women in each covariate category, by age at first childbirth, among women with at least two childbirths[a]

Age at first childbirth (years)	≤19	20-24	25-29	30-34	≥ 35
Total number of women	4020	11,099	4560	772	133
BMI (kg/m^2)					
< 25	43	44	47	47	36
25-29.9	38	38	37	36	42
≥ 30	18	18	16	18	21
Physical activity per week					
< 1hour hard	81	80	77	79	78
1-2 hours hard	14	15	18	18	12
≥ 3hours hard	5	5	5	4	10
Cigarette smoking					
never	26	39	48	52	52
daily former	26	28	30	30	32
daily current	48	33	22	19	17
Education (years)					
≤ 9	51	40	26	28	33
10-12	50	44	38	29	23
≥ 13	7	15	36	43	44
HADS score					
0-4	30	34	36	36	28
5-9	34	35	36	34	41
10-14	20	18	18	18	14
15-19	9	8	6	8	12
20-39	7	5	4	5	6

HADS: Hospital anxiety and depression scale

[a]Among women who were not pregnant when information was collected

Supplementary Table 3 Prevalence of chronic LBP by number of childbirths and pregnancy status, with and without adjustment for HADS

	PR (95 % CI) with standard adjustment[a]	PR (95 % CI) among individuals with known HADS, with standard adjustment[a]	PR (95 % CI) with standard adjustment[a] and adjustment for HADS
Number of women included in the analysis	25,444	22,116	22,116
Number of childbirths			
0	1.00 (reference)	1.00 (reference)	1.00 (reference)
1	1.11 (1.01-1.22)	1.15 (1.04-1.28)	1.12 (1.01-1.24)
2	1.12 (1.03-1.22)	1.14 (1.04-1.25)	1.11 (1.01-1.22)
3	1.17 (1.07-1.27)	1.19 (1.08-1.31)	1.15 (1.05-1.27)
4	1.21 (1.10-1.34)	1.20 (1.08-1.35)	1.16 (1.04-1.29)
5	1.11 (0.97-1.27)	1.18 (1.02-1.36)	1.15 (1.00-1.32)
≥ 6	1.07 (0.91-1.27)	1.05 (0.87-1.28)	1.02 (0.85-1.23)
Currently pregnant[b]	0.80 (0.63-1.00)	0.78 (0.61-1.00)	0.79 (0.62-1.01)
P for categorical effect[c]	0.008	0.017	0.08
P for linear trend[d]	0.08	0.20	0.27

LBP: Low back pain; HADS: Hospital anxiety and depression scale; PR: Prevalence ratio; CI: Confidence interval

[a]Adjustment for age, BMI, physical activity, education, smoking

[b]Regardless of the number of previous childbirths

[c]Among all women who were not currently pregnant

[d]Among women with at least one child

Supplementary Table 4 Prevalence of chronic LBP by age at first childbirth, in women with at least two childbirths, with and without adjustment for HADS

	PR (95 % CI) with standard adjustment[a]	PR (95 % CI) among individuals with known HADS, with standard adjustment[a]	PR (95 % CI) with standard adjustment[a] and adjustment for HADS
Number of women included in the analysis	18,384	15,770	15,770
Age at first childbirth (years)			
≤ 19	1.36 (1.25-1.49)	1.32 (1.20 -1.45)	1.27 (1.16-1.40)
20-24	1.18 (1.10-1.27)	1.17 (1.09-1.27)	1.18 (1.09-1.27)
25-29	1.00 (reference)	1.00 (reference)	1.00 (reference)
30-34	1.04 (0.89-1.20)	1.01 (0.86-1.19)	0.98 (0.84-1.15)
≥ 35	1.23 (0.91-1.66)	1.15 (0.82-1.63)	1.10 (0.78-1.55)
P for categorical effect	< 0.001	< 0.001	< 0.001
P for linear trend	< 0.001	< 0.001	< 0.001
P for quadratic effect	< 0.001	0.001	0.025

LBP: low back pain; HADS: Hospital anxiety and depression scale; PR: prevalence ratio; CI: confidence interval

[a]Adjustment for age, number of children, age at first or last delivery, BMI, physical activity, education, smoking
